# Reevaluating the Utility of Liver Biopsies in Patients With Melanoma

**DOI:** 10.7759/cureus.94149

**Published:** 2025-10-08

**Authors:** Jacob Sapell, Mustafa Al-Roubaie, Altan Ahmed, Hakob Kocharyan

**Affiliations:** 1 Interventional Radiology, University of South Florida Morsani College of Medicine, Tampa, USA; 2 Interventional Radiology, Moffitt Cancer Center, Tampa, USA

**Keywords:** confounding factors, decision-support tools, interventional radiology, malignant melanoma metastasis, metastatic liver lesion

## Abstract

Introduction

This study explores whether some patients with a prior melanoma diagnosis and new liver lesions can be presumed to have metastatic melanoma based on clinical suspicion alone, thereby avoiding the need for liver biopsies. The goal is to enable earlier treatment while reducing the risks, costs, and delays associated with invasive biopsy procedures.

Methods

This retrospective cohort study reviewed patients with a history of melanoma who underwent ultrasound- or CT-guided liver biopsies between 2014 and 2023. Patients biopsied for liver toxicity assessment, treatment response, or who underwent only fine-needle aspiration were excluded. Chart review captured biopsy outcomes, metastatic history, presence of solitary or multiple liver lesions, and the presence of confounding cancers (any history of non-melanoma malignancy at the time of biopsy). Statistical analysis included Fisher’s exact test and binomial CIs.

Results

Among 143 patients, 111 (77.6%) had positive biopsy results for melanoma (95% CI: 69.9%-84.2%). Of the 76 with a known history of metastatic melanoma, 63 (82.9%) had positive biopsy results, although this association was not statistically significant (p=0.114). The presence of confounding cancer was significantly associated with melanoma-negative biopsy results (p=6.50×10^-^⁷), with an odds ratio of 0.106 (95% CI (0.038, 0.281)), indicating a markedly lower likelihood of melanoma metastasis in patients with another known malignancy. Solitary versus multiple lesions were also associated with biopsy outcome. Patients with solitary lesions had significantly lower odds of a melanoma-positive biopsy compared to those with multiple lesions (odds ratio=0.32; 95% CI (0.123, 0.837); p=0.015).

Conclusion

The majority of patients with suspected liver metastases were confirmed to have metastatic melanoma on biopsy, suggesting that clinical suspicion may often be accurate in patients with a history of melanoma. Although a known history of metastatic melanoma did not reach statistical significance, the trend may still be clinically relevant. Lesion number was also associated with biopsy outcome, with solitary lesions significantly less likely to yield melanoma-positive results. In contrast, the presence of a confounding cancer significantly reduced the odds of a melanoma-positive biopsy, underscoring the importance of comprehensive oncologic history in diagnostic decision-making. These findings support consideration of a more selective approach to liver biopsy in melanoma patients, but biopsy remains essential in many scenarios. Validation in larger cohorts is needed before clinical practice changes can be made.

## Introduction

Melanoma is an aggressive malignancy with a high propensity for distant metastasis, including to the liver. Liver involvement is particularly common in certain melanoma subtypes, including ocular melanoma, but remains a clinically significant feature of advanced disease across presentations [1,2]. Regardless of subtype, liver metastases are associated with poor prognosis and play a key role in guiding treatment decisions [1]. Life expectancy with malignant melanoma with gastrointestinal tract or liver metastases is virtually six to nine months, but can vary based on the type of melanoma and specific treatments received [3].

The liver’s vascular and immunologic microenvironment supports metastatic colonization through interactions involving Kupffer cells, sinusoidal endothelial cells, and local cytokine signaling. In particular, cytokines such as IL-6 and VEGF promote angiogenesis and immune evasion [4]. Melanoma cells that establish liver metastases possess several survival adaptations, including enhanced antioxidant capacity, resistance to apoptosis, and increased expression of glutathione metabolism and anti-apoptotic proteins such as Bcl-2 [5].

Image-guided liver biopsy remains the standard approach to confirm metastatic disease before initiating systemic therapy. In a large oncologic series of 580 patients, Elsayes et al. reported that 91% of liver biopsies were positive for malignancy, including many patients with melanoma, while approximately 10% of cases revealed unexpected findings or a diagnosis unrelated to the presumed primary tumor [6]. Improvement in biopsy technique, including coaxial core needle systems, has enhanced tissue adequacy while lowering complication and tumor seeding rates [7]. Despite these advances, biopsy introduces procedural risks, potential treatment delays, and occasional nondiagnostic results [8,9].

Although this study did not assess imaging features directly, previous literature has shown that melanoma liver metastases frequently appear hyperintense on T1-weighted MRI due to melanin and hemorrhagic components and often demonstrate strong enhancement during the arterial phase because of their hypervascularity [10]. These imaging characteristics, when considered alongside a known history of melanoma, may allow for a presumptive diagnosis without biopsy in carefully selected clinical scenarios [11].

This study aims to evaluate the diagnostic yield of image-guided liver biopsies in patients with a history of melanoma and to identify specific clinical factors that are associated with biopsy outcomes. In particular, the study examined whether prior metastatic history, the presence of confounding cancers, or lesion number were associated with the likelihood of a biopsy confirming melanoma. These objectives are intended to clarify the situations in which a biopsy may be most informative versus scenarios where it may be avoidable.

## Materials and methods

This retrospective observational study was conducted at a tertiary academic medical center to evaluate the diagnostic yield of image-guided liver biopsies in patients with a history of melanoma. Eligible patients were 18 years or older, had a confirmed diagnosis of melanoma prior to biopsy, and underwent percutaneous image-guided liver biopsy between January 1, 2016, and June 30, 2023. Cases were identified through a search of institutional radiology records, and clinical data were collected through electronic chart review. 

The following variables were collected: patient age, sex, presence of known metastatic melanoma at the time of biopsy, biopsy result, and presence of a confounding cancer, defined as any prior or concurrent diagnosis of a non-melanoma malignancy. Lesion number (solitary vs. multiple) was also recorded. PET avidity and BRAF mutation status were collected when available, but were excluded from analysis due to high rates of missing data.

Patients were excluded if they did not have a prior melanoma diagnosis at the time of biopsy or if the liver biopsy was performed for reasons unrelated to the evaluation of a newly identified liver lesion. Fine-needle aspiration cases were not included, as only image-guided core needle biopsies were captured for this study.

The primary outcome was whether the biopsy was positive for melanoma. Descriptive statistics were used to summarize results. Fisher’s exact test was used to assess associations between categorical variables, and binomial CIs were calculated where appropriate. All patient records were de-identified prior to analysis to protect patient confidentiality.

## Results

A total of 143 patients with a history of melanoma underwent image-guided liver biopsy during the study period. Of these, 111 biopsies (77.6%; 95% CI (69.9%, 84.2%)) were positive for melanoma, and 32 (22.4%; 95% CI (15.8%, 30.1%)) were negative (Table 1).

**Table 1:** Biopsy results for all patients with a history of melanoma.

Biopsy Result for Melanoma	Count	Percentage	95% CI (%)
Negative	32	22.4	[15.8, 30.1]
Positive	111	77.6	[69.9, 84.2]

Among patients with a known history of metastatic melanoma at the time of biopsy, 63 of 76 (82.9%) had positive liver biopsy results. Among those without known metastatic disease, 48 of 67 (71.6%) were biopsy-positive. However, this difference was not statistically significant (Fisher’s exact test, p=0.114).

A history of a confounding cancer, defined as any concurrent or prior non-melanoma malignancy, was associated with a significantly lower likelihood of a positive biopsy. Among 31 patients with a confounding cancer, 13 (41.9%) had biopsy-positive results, compared to 98 of 112 patients (87.5%) without a confounding cancer. This difference was highly significant (Fisher’s exact test, p=6.50×10^-^⁷), with an odds ratio of 0.106 (95% CI (0.038, 0.281)), indicating that the odds of a positive biopsy were approximately 90% lower in patients with a confounding cancer (Table 2).

**Table 2:** Association between biopsy result and history of confounding cancer. Patients with a confounding cancer had significantly lower odds of a melanoma-positive biopsy (Fisher’s exact test p=6.50×10^-^⁷; odds ratio=0.106).

Confounding Cancer	Biopsy Positive for Melanoma	Biopsy Negative for Melanoma	Total	% Positive
Yes	13	18	31	41.9%
No	98	14	112	87.5%

The lesion number was also significantly associated with biopsy outcome. Among the 33 patients with solitary lesions, 20 (60.6%) had biopsy-positive results, compared to 91 of 110 patients with multiple lesions (82.7%). This difference was statistically significant (Fisher’s exact test, p=0.015), with an odds ratio of 0.32 (95% CI (0.123, 0.837)), indicating that the odds of a melanoma-positive biopsy were significantly lower in patients with solitary lesions (Table 3).

**Table 3:** Association between lesion number and biopsy result. Patients with solitary liver lesions were significantly less likely to have melanoma-positive biopsies compared to those with multiple lesions (Fisher’s exact test p=0.015; odds ratio=0.32; 95% CI (0.123, 0.837)).

Lesion Type	Biopsy Positive for Melanoma	Biopsy Negative for Melanoma	Total	% Positive
Solitary	20	13	33	60.6%
Multiple	91	19	110	82.7%

## Discussion

This study examined the diagnostic role of image-guided liver biopsy in patients with a history of melanoma and found that most biopsies confirmed hepatic metastases. Among patients without known metastatic disease, a substantial proportion were biopsy-positive, suggesting that liver lesions in this group often represent true progression. These findings raise the possibility that, in select cases with strong clinical or radiologic suspicion and no competing diagnoses, biopsy may not always be necessary to initiate treatment.

Although the difference in biopsy positivity between patients with and without known metastatic melanoma did not reach statistical significance, the observed trend is clinically relevant. It aligns with prior literature describing the liver as a common site of melanoma metastasis [1,2], including in patients with ocular melanoma, where liver involvement is particularly prevalent [12]. Prior studies of percutaneous liver biopsy in mixed cancer cohorts have reported high diagnostic yields approaching 90%, but also note that up to 10% of cases reveal benign or unexpected non-index tumor diagnoses [6]. This underscores both the strength and limitations of biopsy as a diagnostic tool. 

The presence of a confounding cancer was significantly associated with a lower likelihood of a melanoma-positive biopsy. This finding reinforces the importance of tissue sampling in patients with diagnostic ambiguity. When more than one malignancy is present, liver lesions may be misattributed on imaging alone. In these cases, biopsy remains critical to avoid incorrect treatment selection [13]. This is consistent with recent studies demonstrating that differentiating between metastatic deposits and primary liver cancers, such as hepatocellular carcinoma, can be challenging as imaging features frequently overlap [13].

Lesion number was also significantly associated with biopsy outcome. Patients with solitary liver lesions, 20 of 33 (60.6%), were less likely to have melanoma-positive biopsies than those with multiple lesions, 91 of 110 (82.7%). This finding suggests that the number of liver lesions may serve as an additional clinical factor when considering whether biopsy is warranted. As with confounding cancer, lesion number may contribute to diagnostic confidence in select scenarios. Prior oncologic series have similarly shown that metastatic disease typically presents as multifocal hepatic involvement, whereas solitary lesions are more likely to be benign or unrelated to the known primary tumor [6].

While a biopsy generally carries a high diagnostic yield, it is not without risk. A recent meta-analysis by Thomaides-Brears et al. of more than 64,000 percutaneous liver biopsies reported a major complication rate of 2.4%, with mortality estimated at 0.01%. Hospitalization occurred in 0.65% of cases, major bleeding in 0.48%, and moderate to severe pain in 0.34%. Minor complications were reported in 9.5% of cases, most commonly pain, while technical failure occurred in approximately 0.9%. Younger age and greater disease severity were associated with higher rates of hospitalization and bleeding [9]. Although the absolute risk remains low, these findings highlight that complications are not negligible, particularly in vulnerable patients. In the context of melanoma, where clinical and radiologic features may strongly suggest metastatic disease, weighing the diagnostic benefit of biopsy against the potential for harm remains an important consideration. 

These results should also be interpreted in the context of evolving melanoma therapies. Immunotherapy and targeted agents have transformed the treatment landscape, making histologic confirmation important in some clinical scenarios to guide therapeutic sequencing [14]. For example, the identification of BRAF mutations or confirmation of progression in the setting of immunotherapy can influence the choice and timing of systemic therapy. The results of this study suggest a possible role for a more individualized approach to biopsy decisions in melanoma. A clinical tool that incorporates factors such as prior metastatic history, imaging characteristics, PET findings, and absence of other malignancies could help identify patients in whom biopsy might be safely avoided. While this study was not designed to establish such criteria, the findings provide preliminary support for further investigation.

Limitations of this study include its retrospective design, reliance on electronic medical record documentation, and single-institution setting. Imaging features were not formally assessed, and it is possible that some negative biopsies lacked adequate follow-up to identify false negatives. Data on PET avidity and BRAF mutation status were collected but excluded from analysis due to the low rate of completion. Lesion size was not collected in this study, which may limit the ability to assess its potential impact on diagnostic yield and should be evaluated in future work aimed at developing clinical decision-support tools. These limitations should be considered when interpreting the generalizability of the findings. Additionally, while our findings highlight potential clinical patterns, they are exploratory in nature and not intended to guide immediate changes in biopsy practice.

Liver biopsy frequently confirmed metastatic melanoma in patients with a history of melanoma, including those without previously known distant disease. However, the presence of a confounding cancer and the number of liver lesions significantly influenced biopsy yield. These findings support further exploration into the selective use of liver biopsy based on clinical context and may inform future decision-making tools to help guide practice.

## Conclusions

In this retrospective study, liver biopsy confirmed metastatic melanoma in the majority of patients with a history of melanoma and suspected hepatic involvement. This included many patients without previously known distant disease, suggesting that liver lesions in this population often represent true metastases. However, the presence of a confounding cancer was significantly associated with a lower likelihood of melanoma-positive biopsy, underscoring the importance of histologic confirmation in patients with multiple potential sources of metastasis. Lesion number also influenced diagnostic yield, with solitary lesions less likely to be biopsy-positive than multiple lesions. These findings suggest that certain clinical factors may be associated with diagnostic yield, but biopsy remains essential in many scenarios. Future studies with larger cohorts and integrated imaging or molecular data are needed to develop validated clinical decision-support tools that could eventually help guide when a biopsy might be avoidable.
